# Surgical Management of Gossypiboma: A Case Report

**DOI:** 10.7759/cureus.46797

**Published:** 2023-10-10

**Authors:** Sneha George Teressa, Juhi Saxena, Muhammad Afzal, Bruce Morel

**Affiliations:** 1 General Surgery, BronxCare Health System, New York, USA; 2 General Surgery, St. George’s University School of Medicine, True Blue, GRD; 3 General Surgery, Queens Hospital Center, Queens, USA

**Keywords:** textiloma, surgical management, interventional radiology, surgery, gossypiboma

## Abstract

Gossypiboma is a rarely reported surgical complication and refers to a retained surgical textile in the body after a procedure. The surrounding inflammation and reaction often manifest as acute pain and subsequently require additional surgery. We report the case of a 33-year-old female who presented with acute abdominal pain one month after undergoing an exploratory laparotomy secondary to a gunshot wound in her home country. A diagnosis of retained foreign body was made with radiological imaging and confirmed upon the retrieval of two surgical sponges after the operation. Due to the high morbidity and mortality as well as increased healthcare costs, strict protocols must be followed to avoid such outcomes.

## Introduction

The term “gossypiboma” refers to a retained surgical sponge or textile in the human body. Historically, gossypiboma is a combination of the Latin word “gossypium” meaning cotton and the Swahili word “boma” meaning “place of concealment” [1]. Although this is a well-established radiological term, there is a limited discussion of gossypiboma in the surgical literature. Gossypibomas are the most common retained surgical items and are estimated to occur at a rate of 1 in every 1,000-1,500 intra-abdominal operations [2]. Understanding the true prevalence of this phenomenon poses great challenges due to medicolegal consequences [3]. These cases are considered a medical never event generally due to iatrogenic error and are greatly underreported in the literature [3].

## Case presentation

A 33-year-old female presented to the Emergency Department (ED) of our institution with a one-day history of progressively worsening left lower quadrant and right flank pain. She had suffered a gunshot wound to the abdomen one month ago in a foreign country for which she underwent emergency exploratory laparotomy, right nephrectomy, and ileo-colectomy with primary anastomosis with loop ileostomy. On postoperative day 10, she had chills and diffuse abdominal pain which was determined to be a contained anastomotic leak in the foreign hospital. She was treated conservatively for four weeks with intravenous antibiotics and was discharged with symptomatic improvement. She traveled to the United States once she felt relatively better. Her symptoms had not completely resolved and she presented to our ED with worsening abdominal pain.

On a physical examination upon admission, the patient was afebrile, blood pressure was 101/66 mmHg, heart rate was 70 beats/minute, and respiratory rate was 18 breaths/minute. The abdomen was soft with tenderness in the left lower quadrant and right flank without guarding. Right-sided costovertebral angle tenderness was also present. The rest of her physical examination was unremarkable.

Investigations

Upon arrival, a complete blood count revealed a normal white blood cell count but a decreased hemoglobin value of 10.3 g/dL and a hematocrit value of 32.6% (Table 1). Urinalysis was significant for blood and small leukocyte esterase in the urine. Blood culture was positive for methicillin-resistant *Staphylococcal epidermidis*.

**Table 1:** Complete blood count laboratory values upon admission. L: low

Component	Reference range	Patient values
White blood cells	4.80–10.80 × 10^3^/µL	10.48
Red blood cells	4.20–5.40 × 10^6^/µL	3.61 (L)
Hemoglobin	12.0–16.0 g/dL	10.3
Hematocrit	37.0–47.0%	32.6 (L)
Neutrophil	44.9–70.0%	61.5

**Figure 1:**
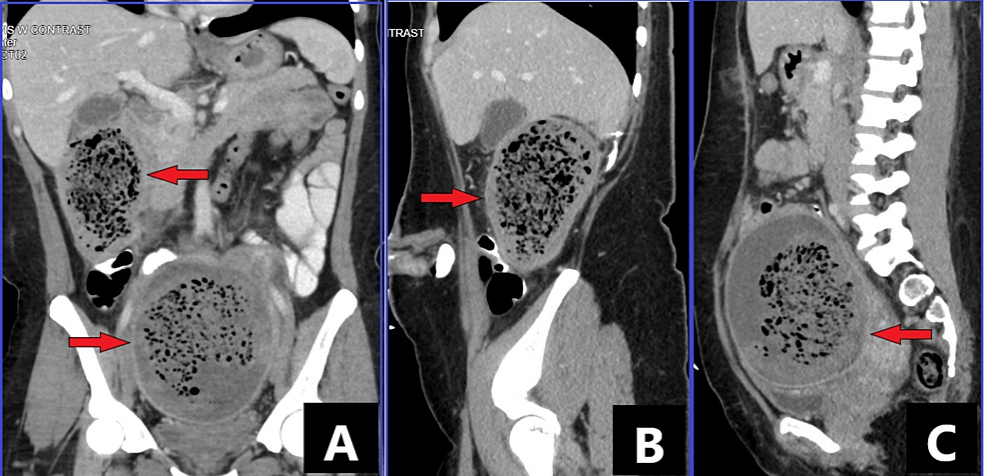
Peripherally enhancing, contained, heterogenous areas with both hypodense and fluid components (arrows) seen on CT scan of the abdomen and pelvis in the coronal (A) and sagittal view (B, C).

Urgent CT with contrast showed a large mildly thick-walled complex heterogeneous collection of air and fluid surrounding the right nephrectomy bed measuring 9.3 cm × 8.2 cm × 12.8 cm (Figures 1A, 1B). A second, similarly appearing large mildly thick-walled collection was also noted in the pelvis anterior to the uterus and superior to the bladder measuring 13.5 cm × 11.7 cm × 14.5 cm (Figures 1A, 1C). The pelvic collection was also compressing on the left distal ureter leading to mild left hydronephrosis and moderate hydroureter. Empiric coverage was started with piperacillin-tazobactam (Zosyn) and metronidazole (Flagyl).

Treatment

Initially, the patient was managed with intravenous fluids and broad-spectrum antibiotics. In addition, interventional radiology was consulted and attempted a minimally invasive procedure of CT-guided drainage with a percutaneous catheter. Drainage of the pelvic collection yielded 600 mL of purulent fluid. However, the drainage of the collection in the right renal fossa was unsuccessful after multiple attempts.

**Figure 2:**
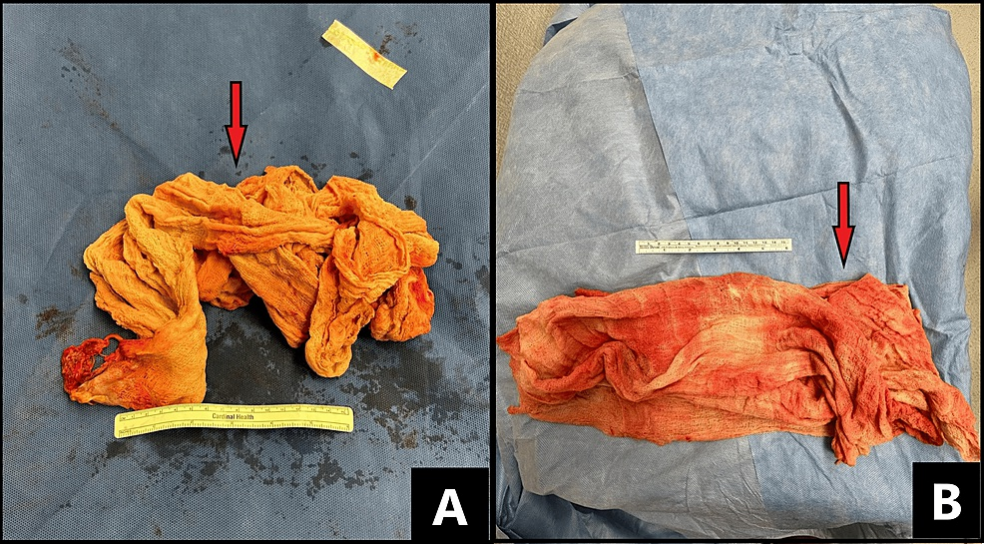
Surgical sponges recovered (arrows) from the right renal fossa (A) and pelvis (B).

On day two of admission, the patient was subsequently taken to the operating room for an exploratory laparotomy. Before the exploratory laparotomy, cystoscopy and left ureteral stenting were performed by a separate Urology team. Intraoperatively, a single white lap pad was found in the right nephrectomy bed (Figure 2A). Using finger dissection, it was bluntly removed from adhesions and a thick capsule. It was measured to be 150 cm and full of fibrinous purulent fluid. A second white lap pad measuring 150 cm covered in purulent material was recovered from the pelvic area (Figure 2B). Body fluid culture returned positive for rare *Enterococcus faecalis* and extended-spectrum *Escherichia coli*. During the procedure, two 10 French Jackson-Pratt (JP) surgical drains were left in the abdominal cavity. No operative complications were encountered.

Postoperatively, the patient was initially treated with piperacillin-tazobactam and ceftriaxone, but this was adjusted to meropenem 1,000 mg every eight hours after culture sensitivities showed resistance. There were no immediate postoperative complications, and the patient was subsequently discharged on postoperative day five with two JP drains and close follow-up.

## Discussion

Gossypibomas have a variable onset and presentation. Gossypibomas have been reported most commonly after abdominal and gynecological surgeries [1]. The timeline of symptom presentation can vary from weeks to months after surgery. Clinically, physicians should have a suspicion of gossypiboma in patients with nonspecific complaints, fever, nausea, vomiting, abdominal pain, and other symptoms with a history of previous abdominal surgery [2,3].

Generally, the pathophysiology has been described as two distinct inflammatory reactions in the body. If there is an exudative reaction, the patient may present with an acute abscess formation. Alternatively, a granulomatous reaction may cause asymptomatic abdominal adhesions and aseptic fibrous tissue formation [1,4]. This eventually will result in the encapsulation and development of a foreign body granuloma that can be identified on imaging [4]. In our patient, the two retained surgical sponges created a granulomatous reaction in the abdominal cavity, which appeared completely walled off on imaging.

Imaging modalities such as CT and X-ray do not have consistent radiological features to lead to prompt diagnosis. Gauze that is radiopaque may have a heterogeneous spongiform appearance with air bubbles or calcifications [4]. Over time calcium deposits generate a “calcified reticulate rind” sign that is considered a characteristic finding for the diagnosis of gossypibomas [5]. Gossypibomas vary from cystic lesions to tumor-mimicking masses [1]. Ultrasound has been used as an adjunct to help delineate the mass as solid versus fluid in nature when pseudotumor syndrome is suspected [6].

In this case, the patient retained a surgical sponge after her first emergency laparotomy done post-gunshot wound in another country. It is unique in the aspect that there were two separate, recovered surgical sponges in two distinct locations. In many resource-limited foreign countries, there is no availability of radiopaque sponges. This makes imaging to aid in a diagnosis much more challenging. From a public health perspective, gossypiboma is an avoidable potentially fatal surgical complication. Depending on the size of the gauze and duration in the body, the mortality rate has been reported as 11-35% [4].

It is crucial to consider known risk factors, including emergency surgeries, forgoing the preoperative sponge count, staff changes during the procedure, inexperienced staff, and postoperative high body mass index [7]. During trauma surgeries with extensive bleeding, surgeons may utilize more gauze compared to planned elective surgery. Mitigating these risk factors as much as possible, especially in the setting of a fast-paced or extensive surgery, is imperative. Having a greater availability and accessibility to radiopaque gauze may aid in decreasing the occurrence of this complication. The American College of Surgeons recommends that it is the responsibility of the entire surgical team to locate missing items and endorses an optimal surgical environment with focused operative tasks [3]. The use of a checklist as well as institutional standardized policies can be implemented to aid in the reduction of errors.

## Conclusions

Although rare, postoperatively retained foreign bodies in the abdomen remain underreported and represent a serious surgical issue that must be addressed acutely. Its variable presentation can pose a diagnostic dilemma to clinicians. However, identifying risk factors is important and can lead to prompt intervention. CT scan remains the standard imaging modality to aid diagnosis. Some measures that can be taken to reduce the likelihood of this occurring are verifying surgical instrument count, standardizing intraoperative staff hand-off, and encouraging open communication. This report highlights that retained foreign bodies are a continuous underreported medical problem. A gossypiboma must be considered in patients who underwent emergent surgical procedures. Further research must be conducted on optimal operating room communication and environment to prevent such errors.
